# Metagenome-Assembled Genome of a Putative Methanogenic *Methanosarcina* sp. Strain Enriched from Terrestrial High-CO_2_ Subsurface Sediments

**DOI:** 10.1128/mra.01039-22

**Published:** 2022-11-02

**Authors:** Zeyu Jia, Daniel Lipus, Alexander Bartholomäus, Oliver Burckhardt, Megan Sondermann, Dirk Wagner, Jens Kallmeyer

**Affiliations:** a GFZ German Research Centre for Geosciences, Section Geomicrobiology, Potsdam, Germany; b University of Potsdam, Institute of Geoscience, Potsdam, Germany; Portland State University

## Abstract

A metagenome-assembled genome (MAG), named *Methanosarcina* sp. strain ERenArc_MAG2, was obtained from a 3-month-old H_2_/CO_2_ atmosphere enrichment culture, originally inoculated with 60-m deep drill core sediment collected from the tectonic Eger Rift terrestrial subsurface. Annotation of the recovered draft genome revealed putative archaeal methanogenesis genes in the deep biosphere.

## ANNOUNCEMENT

High-frequency earthquake swarms and the migration of CO_2_-dominated gases from subcrustal magmatic fluids make the Eger Rift’s Cheb Basin (1) a rare and scientifically relevant subsurface site to study microbial behavior and biological-geological (bio-geo) interactions (2–4). Seismic release of H_2_ likely promotes the production of biogenic methane, highlighting the role of methanogenic *Archaea* in this ecosystem (5).

Native microbial communities were enriched from this ecosystem utilizing sediment and rock samples from a 240-m-long drill core recovered from the Hartusov Mofette Field (Czech Republic, 50.13 N 12.46 E). Approximately 5 g of sample material from 8 different depths (46 m to 230 m) was incubated in slurries using DSMZ mineral media 81 for chemolithotrophic growth under CO_2_/H_2_ headspace at 16°C for 3 months. For the metagenome-assembled genome (MAG) reported here, DNA was extracted from an enrichment culture inoculated with 60-m-deep mudstone drill core sediments with the FastDNA isolation kit (MPBio, Irvine, CA), prepared using the rapid barcoding sequencing kit (Oxford Nanopore Technologies [ONT], Oxford, UK), and cleaned up using AMPure XP beads (Beckman Coulter, Pasadena, CA), removing small DNA fragments. The resulting library was sequenced using the MinION platform (ONT) with flow cell quality 9.4.1 (on FLO-MIN106) for 72 h, generating 372,894 quality reads (passing default quality control) with an *N*_50_ value of 3,980 bp. Sequencing raw data were basecalled and demultiplexed using super high accuracy with guppy v4.4.2 + 9623c1626 (ONT). Default parameters were used for all software unless otherwise specified. Assembly with Flye v2.8.2-b1689 (6) (parameters: –plasmid–meta) resulted in 419 contigs with an *N*_50_ value of 111,337 bp. Assembled contigs were binned with MaxBin 2.0 (7). Bin quality was assessed using the lineage_wf workflow, and full-length 16S rRNA gene sequences were recovered using the ssu_finder tool of CheckM v1.0.13 (8).

The recovered MAG had a size of 4,530,822 bp across 18 contigs with a GC content of 40.1%, an *N*_50_ value of 512,509 bp, and an average coverage of 98×. The genome was found to be 93.4% complete and 4.9% contaminated (8). Taxonomic assessment using GTDB-Tk v1.5.0 (9) classified the recovered MAG as *Methanosarcina*. A comparison to other *Methanosarcina* genomes by calculating the average nucleotide identity (ANI) using the JSpeciesWS tool (accessed March 2022) (10) suggested the MAG to share the most nucleotide-level genomic similarity with Methanosarcina barkeri MS (ANI by BLAST [ANIb], 81.62%; ANI by MUMmer [ANIm], 85.16%). A full-length 16S rRNA gene sequence comparison (1,472 bp) using NCBI BLAST (11) revealed 98.4% sequence identity to Methanosarcina siciliae strain T4/M.

Annotation via NCBI Prokaryotic Genome Annotation Pipeline (PGAP) (12) resulted in the discovery of putative hydrogenotrophic, methanogenesis genes, including operons encoding methyl coenzyme M reductase alpha subunit (EC 2.8.4.1, locus tag NHB15_06240) and N5-methyltetrahydromethanopterin:coenzyme-M-methyltransferase subunit A (EC 2.1.1.86, locus tag NHB15_17870).

### Data availability.

The MAG was deposited at NCBI with the accession number JAMXHR000000000 under BioProject PRJNA832091. Raw reads are accessible via the accession SRX15925126. The 16S rRNA gene sequence is available at NCBI under the accession number ON862867.
